# Peroxisome Proliferator-Activated Receptors as a Therapeutic Target in Asthma

**DOI:** 10.1155/2020/8906968

**Published:** 2020-01-14

**Authors:** Oxana Yu. Kytikova, Juliy M. Perelman, Tatyana P. Novgorodtseva, Yulia K. Denisenko, Viktor P. Kolosov, Marina V. Antonyuk, Tatyana A. Gvozdenko

**Affiliations:** ^1^Vladivostok Branch of Far Eastern Scientific Centre of Physiology and Pathology of Respiration, Institute of Medical Climatology and Rehabilitative Treatment, Vladivostok, Russia; ^2^Far Eastern Scientific Center of Physiology and Pathology of Respiration, Russian Academy of Sciences, Blagoveshchensk, Russia

## Abstract

The complexity of the pathogenetic mechanisms of the development of chronic inflammation in asthma determines its heterogeneity and insufficient treatment effectiveness. Nuclear transcription factors, which include peroxisome proliferator-activated receptors, that is, PPARs, play an important role in the regulation of initiation and resolution of the inflammatory process. The ability of PPARs to modulate not only lipid homeostasis but also the activity of the inflammatory response makes them an important pathogenetic target in asthma therapy. At present, special attention is focused on natural (polyunsaturated fatty acids (PUFAs), endocannabinoids, and eicosanoids) and synthetic (fibrates, thiazolidinediones) PPAR ligands and the study of signaling mechanisms involved in the implementation of their anti-inflammatory effects in asthma. This review summarizes current views on the structure and function of PPARs, as well as their participation in the pathogenesis of chronic inflammation in asthma. The potential use of PPAR ligands as therapeutic agents for treating asthma is under discussion.

## 1. Introduction

Asthma is a commonly occurring chronic inflammatory disease with high variability due to the influence of genetic and environmental factors [1, 2]. The incidence of asthma in industrialized countries is steadily growing, and more than 100 million people have been estimated to suffer from this disease by 2025 [3]. Despite the effectiveness of traditional methods of asthma treatment, a number of patients have exacerbations and progressive deterioration of pulmonary function. It can be accounted for by the heterogeneity of the disease and the complexity of pathogenetic mechanisms.

The disorders of the immunoreactivity, fatty acid composition of cell membranes, and the imbalance between the substrates for the synthesis of pro- and anti-inflammatory mediators are important for the regulation of persistence and resolution of inflammation in the bronchopulmonary system. Therefore, the possibilities for the regulation of immune and lipid metabolism disorders in asthma are being actively studied. Various immune mechanisms involved in asthma pathogenesis determine the type of inflammation and form the endotypes of the disease. However, even the determination of asthma endotypes, which is aimed at choosing effective therapeutic approaches to reduce the symptoms of the disease and improve the quality of life of patients, does not make it possible to suppress chronic inflammation in the bronchopulmonary system. It indicates that there are subendotypes that include disturbances at different structural levels. For example, chronic inflammation in asthma can be inhibited both by the intracellular signaling pathways and by the activity of inflammatory genes [4]. The change in the activity of some receptors affects the functioning of others and contributes to the development of chronic inflammation. Thus, the correction of disorders in the signaling pathways involved in immune response and lipid metabolism in asthma provides many opportunities to improve the methods of the treatment of the disease.

Peroxisome proliferator-activated receptors (PPARs) are involved in the regulation of inflammatory reactions and lipid metabolism. The anti-inflammatory properties of PPARs are mainly achieved by inhibiting nuclear factor-kappa B (NF-*κ*B) which, in turn, is the proinflammatory nuclear transcription factor. The relationship between PPARs and NF-*κ*B is an important object of the current studies, since it may serve as a target for development of strategies to control the activity of the inflammatory process [5, 6]. The interactions between PPARs, NF-*κ*B, and toll-like receptors (TLRs) are of great interest [7]. Along with the anti-inflammatory mechanism of action of PPARs, the proinflammatory activity of some isoforms of PPARs is also being studied. For example, PPAR*γ* is considered a mediator of interactions between dendritic and T cells in the development of type 2 (or T2) inflammation [8].

PPARs are key metabolic regulators of whole-body energy metabolism as well. PPARs regulate the transcription of eicosanoid genes and fatty acids (FAs) and are associated with transcriptional activation of peroxisomal FA *β*-oxidation. Some enzymes involved in carbohydrate and lipid metabolism are directly regulated by PPARs through their interaction with peculiar elements of response in their gene promoters [9]. The anti-inflammatory effect of PPARs is related to lipid metabolism.

The foregoing makes PPARs an extremely topical area with many unresolved problems concerning the signaling pathways of these receptors in asthma.

PPARs are stimulated by a wide range of natural and synthetic activators resulting in the normalization of lipid metabolism, as well as in the modulation of proinflammatory response and the prevention of their excessive activation. Close attention currently focuses on synthetic PPAR agonists (fibrates and thiazolidinediones). The effectiveness of their action in asthma is extensively studied and, according to some authors, is much higher in combination with corticosteroids [10–13]. At the same time, negative reactions to the application of synthetic PPAR agonists and their effect on different PPAR isoforms should be taken into account. In our opinion, the study of therapeutic efficiency of natural PPAR agonists (polyunsaturated fatty acids (PUFAs), eicosanoids, endocannabinoids, and endogenous specialized proresolving mediators (SPMs)) with a lower number of side effects than that of synthetic ones may be an incentive to develop a new combined therapy of asthma.

Thus, the complexity of mechanisms that determine the heterogeneity of asthma results in low effectiveness in treating the disease. This review summarizes modern views on the structure and functions of PPARs, as well as their participation in the pathogenesis of chronic inflammation in asthma. The possibility to use PPARs as a therapeutic target in asthma has been described.

## 2. Asthma

The pathogenesis and clinical manifestations of asthma are characterized by heterogeneity, which is expressed by the presence of various endotypes and phenotypes of the disease [14]. The asthma phenotype combines a set of clinical and physiological signs of the disease, as well as pathobiological features associated with clinical manifestations, which make it possible to isolate asthma phenotypes at the molecular level [15]. An asthma endotype is characterized by the presence of a specific pathobiological marker or mechanism (for example, sIgE levels, the number of eosinophils in induced sputum, and fractional expired nitric oxide (FeNO)), which may be a target for pathogenetic therapy of the disease [15]. The term “asthma” is recognized as a diagnosis that combines several endotypes and various phenotypes, each of which being manifested by wheezing, coughing, shortness of breath, chest tightness, decreased expiratory airflow, hyperreactivity, airway remodeling, and mucus hyperproduction [16]. It is known that even within the same endotype, patients may have both different degrees of disease severity and a response to the treatment [17]. Phenotypes of the disease can also change with time [18].

Stratification of patients by the inflammatory endotype is recognized as the basis for developing asthma treatment strategies [16]. Both innate and adaptive immunity play an important role in the immunological mechanisms of asthma. A number of patients with asthma have an imbalance in the T lymphocyte system that is characterized by a predominance of T helper 2 cell (Th2) response over T helper 1 cell (Th1) one. This is the basis for the isolation of the Th2-high (eosinophilic) asthma endotype. However, the development of inflammation includes not only Th2 cells and eosinophils but also type 2 innate lymphoid cells (ILC2), IgE-secreting B cells, basophiles, and dendritic and mast cells. Therefore, Th2-high inflammation is also referred to as type 2 (or T2) inflammation, to take into account the role of other immune cells involved in Th2 airway inflammation [16]. Type 2- (T2-) high type of inflammation is asthma, characterized by eosinophilic inflammation both with and without the development of an allergic component (early-onset allergic asthma, late-onset eosinophilic asthma, and aspirin-exacerbated respiratory disease (AERD)). The main cells associated with type 2- (T2-) high 2 asthma are eosinophils, due to the participation in the formation of various inflammatory mediators: IL-13, IL-5, chemokines, and cysteinyl leukotrienes, which are the powerful bronchoconstrictors. The inflammation in the bronchial walls in patients with asthma type 2- (T2-) high 2 results in an increase in the basophile number. Basophile and mast cell activation promotes the histamine and prostaglandin production, which causes vasodilatation and increased vascular permeability [19].

An important role in the development of asthma belongs to the activation of bronchial epithelial cells and the induction of cytokine production, such as IL-33, IL-25, and thymic stromal lymphopoietin (TSLP). Alarmins (IL-33, IL-25, and TSLP) activate ILC2, producing IL-5 and IL-13, and, accordingly, enhance early type 2 immune response [20]. In addition, alarmins activate dendritic immune cells, which are necessary for the absorption, transport, and recognition of antigens to T cells.

At the same time, some patients without atopy and allergy do not have Th2-type inflammation (non-T2-high (Th2-low) (noneosinophilic) endotype) [21]. This phenotype combines clinical features such as obesity, smoking, and age. Asthma in such patients is not induced by allergens, but it is the etiological factors such as infections, cigarette smoke, and environmental pollution that cause asthma. In the development of the non-T2-high (T2-low) endotype, a significant role belongs to the dysregulation of the innate immune response, which causes neutrophilic inflammation. The progress of the non-T2-high (T2-low) endotype is related to L-17, which can stimulate the occurrence of neutrophilic airway inflammation. CCR6-expressing CD4+ T cells (T helper 17 cells (Th17)) produce IL-17A, IL-17F, IL-22, IL-8, and IL-6, involved in the activation of neutrophils and macrophages. Neutrophilic inflammation is associated with the formation of hypersensitivity and remodeling of the respiratory tract in nonatopic asthma. This type of inflammation is accompanied by a decrease in steroid sensitivity, and the Th17 endotype is characteristic of steroid-resistant asthma.

Recently, a mixed Th2/Th17 endotype caused by differentiation of Th2 cells into double-positive Th2/Th17 cells has been described [22]. Thus, most of the asthma endotypes corresponding to nonatopic asthma are related not only to allergies [23].

The heterogeneity of asthma is based on the complex immune mechanisms. The classifications of phenotypes and endotypes of asthma still develop in connection with the identification of new cells involved in the pathogenesis and contributing to the clinical manifestations of the disease.

Along with immune imbalance, disorders in the fatty acid composition of cell membranes and the synthesis of lipid mediators involved in the resolution of inflammation also promote chronic inflammation in the bronchopulmonary system [24, 25]. The functions of immune cells (secretion, chemotaxis, and microorganism sensitivity) depend on the state of the lipid structure of the cell membrane [26]. The effect of lipid metabolism disorders on the immunological reactivity of the organism is currently being studied and is characterized by contravention data indicating both the development of metabolic immunosuppression and the activation of the immune system [27, 28]. For example, inhibition of stearoyl-coenzyme A desaturase (SCD) in mice contributed to the development of airway hyperresponsiveness, while SCD1 gene expression was suppressed in bronchial epithelial cells in patients with asthma due to the effects of IL-4 and IL-13 [29]. The participation of lipids in the regulation of immunity and the inflammatory process in asthma results from the ability of PUFAs to turn into powerful inflammatory mediators. In particular, the release of proinflammatory eicosanoids, such as leukotrienes (LTs) and thromboxanes (Txs), which are lipoxygenase and cyclooxygenase products, respectively, of the oxidation of arachidonic acid (AA), is observed at the stage of the acute inflammatory process. Eicosanoids play a key role in the inflammatory process. They initiate the acute inflammatory process necessary to activate immune cells and the synthesis of proinflammatory messengers. Cytosolic phospholipase A2 *α* (cPLA2*α*) is of importance for the production of eicosanoids and participates in the pathogenesis of asthma [30]. The importance of cysteine leukotrienes and prostaglandin D2 in asthma pathogenesis has been established. They are proinflammatory mediators and powerful bronchoconstrictors, causing hyperreactivity and bronchial swelling. The classical inflammation is characterized by the transition from the stage of acute inflammation to the stage of the resolution of the inflammatory process. A key role in this process belongs to proresolving lipid mediators (resolvins (Rvs), lipoxins (LXs), protectins (PDs), and maresins (MaRs)). They have anti-inflammatory and cytoprotective effects resulting from the impact on t5he resolution of the inflammatory response through the involvement of the immune system [31, 32]. The result of their action is the inhibition of chemotaxis and migration of macrophages and neutrophils to the inflammatory area, the blockade of lipid peroxidation processes, the activation of NF-*κ*B, and the inhibition of the synthesis of proinflammatory cytokines.

Thus, the heterogeneity of asthma consists of many components, the most important of them being the immune mechanisms. The immune reactivity of the body in asthma is affected by lipid metabolism disorders and synthesis of proinflammatory and proliferating lipid mediators. Intracellular modulators of inflammatory reactions and lipid metabolism at the gene level are nuclear transcription factors, among which an important role in the pathogenesis of asthma belongs to peroxisome proliferator-activated receptors. The discovery of new properties of PPARs and their ligands will contribute to the development of modern asthma treatments.

## 3. Peroxisome Proliferator-Activated Receptors

**Figure 1:**
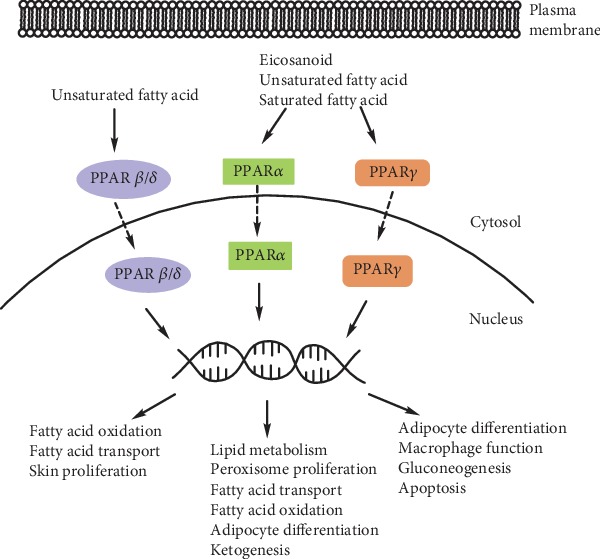
Isoforms of PPARs and their biological effects.

PPARs are members of the superfamily that includes 48 hormonal nuclear transcription factors responsible for metabolism and energy homeostasis of the cell [33]. PPARs play an important part in the regulation of energy homeostasis of the body, cellular processes (differentiation, proliferation, and apoptosis), and immune and inflammatory reactions. Three isoforms of PPARs are known: PPAR*α* (NR1C1), PPAR*β*/*δ* (NR1C2), and PPAR*γ* (NR1C3) (Figure 1).

All isoforms have approximately similar homology and consist of a DNA-binding domain at the N-terminus and a ligand-binding domain (LBD) at the C-terminus [34].

At the same time, the divergence of this LBD domain in isoforms is 20%; thereby, isoforms are characterized by different reactions.

PPAR*α* is expressed primarily in the liver, kidney, heart, skeletal muscle, and brown adipose tissue, as well as in epithelial cells, macrophages, lymphocytes, and dendritic cells [35]. This isoform stimulates the expression of enzyme genes involved in *β*-oxidation and regulates the metabolism of lipids, carbohydrates, and amino acids [36]. PPAR*α* reduces triglycerides and increases the level of high-density lipoproteins in blood plasma. This receptor is activated by unsaturated fatty acids, eicosanoids, and lipid-lowering drugs. PPAR*α* reduces the production of proinflammatory mediators (tumor necrosis factor-*α* (TNF-*α*), IL-1, IL-6, and IL-8) and modulates the expression of adhesive and chemotactic molecules. Activated PPAR*α* can induce the production of anti-inflammatory agents (IL-10), and this fact confirms its modulating effect on the inflammation activity.

PPAR*β*/*δ* (other names PPAR*δ*, PPAR*β*, hNUC1, and FAAR) is expressed in all organs and tissues. The highest expression of PPAR*β*/*δ* is marked in the brain, liver, skin, adipose tissue, and skeletal muscle. PPAR*β*/*δ* is involved in the oxidation of fatty acids, normalizes plasma lipids, regulates blood glucose, and increases cells' sensitivity to insulin, preventing the development of obesity [37]. PPAR*β*/*δ* activators have been put forward to treat metabolic diseases. In addition, PPAR*β*/*δ* abundantly secreted by keratinocytes are active in wound healing [38].

There are three types of PPAR*γ*: PPAR*γ*1, PPAR*γ*2, and PPAR*γ*3. PPAR*γ*1 is expressed in almost all tissues and cells, PPAR*γ*2 is expressed mainly in adipose tissue, and PPAR*γ*3 is expressed in macrophages, the colon and spleen, and white adipose tissue. This receptor is a significant regulator of cellular homeostasis and energy metabolism [34, 39]. In adipose tissue, PPAR*γ* controls adipogenesis and lipid breakdown and increases the cells' sensitivity to insulin. PPAR*γ* promotes lipogenesis during anabolism by acting on the adipose tissue. Besides, this receptor is a participant in inflammatory reactions [4] and a regulator of the immune system in the lungs [10, 40]. The binding of specific ligands to PPAR*γ* regulates the transcription of target genes and inhibits the activation of immune cells and the expression of inflammatory factors [8]. PPAR*γ* and its ligands promote apoptosis of neutrophils and prevent the interaction of platelets and leukocytes. This PPAR isoform activates M2 macrophages and phagocytosis [41].

All PPAR isoforms are mainly expressed in the pulmonary epithelium, endothelium, dendritic cells, eosinophils, fibroblasts, and macrophages and have a dominating role in the homeostasis of the bronchopulmonary system [42]. All isoforms of PPARs are involved in the regulation of lipid metabolism, with its violation being one of the most important mechanisms in the asthma pathogenesis. Such wide expression of PPARs in the bronchopulmonary system, their anti-inflammatory properties, and their ability to regulate lipid metabolism are of particular interest, because the receptors may be a prospective therapeutic target for asthma treatment [4, 10].

## 4. General Mechanisms of Action of Peroxisome Proliferator-Activated Receptors

**Figure 2:**
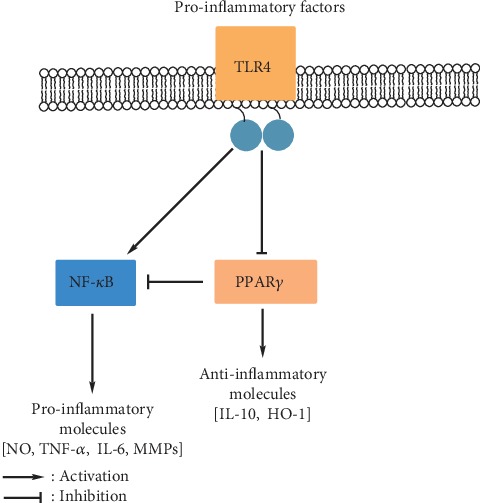
Crosstalk between PPARs and TLRs and NF-*κ*B. HO-1: heme oxygenase 1; NO: nitric oxide; MMPs: matrix metalloproteinases.

The mechanism of PPAR action is primarily characterized by the anti-inflammatory effect. It can be accounted for by the ability of PPARs to regulate the expression of inflammatory genes, as well as the transrepression of proinflammatory genes through the interaction with the p65 subunit of NF-*κ*B (Figure 2).

In interacting with agonists, PPARs translocate to the nucleus and make a heterodimer with retinoid X receptor *α* (RXR*α*) [43]. As a result, genes encoding insulin-1 and insulin-2 receptors (IRS-1 and IRS-2), as well as TNF-*α*, NF-*κ*B, and activator protein-1 (AP-1), are expressed [44]. PPARs inhibit many transcription factors possessing a proinflammatory action: NF-*κ*B, AP-1, protein C/EBP (CCAAT/enhancer-binding protein), and signal transducers and activators of transcription (STAT). The relationships between PPARs and NF-*κ*B are of special interest [5, 6, 37, 45, 46].

Members of the NF-*κ*B family are RelA (p65), RelB, c-Rel, p50/p105 (NF-*κ*B1), and p52/p100 (NF-*κ*B2). As one of the most important messengers, NF-*κ*B stimulates gene expression in response to proinflammatory stimuli, including the expression of mitochondrial genes [47]. An inactive form of NF-*κ*B is located in the cell protoplasm, where it is associated with the NF-*κ*B inhibitor protein (I*κ*B). NF-*κ*B activation is regulated by tumor necrosis factor receptors (TNFR), an interleukin-1 receptor (IL-1R), and TLRs. Besides, DNA damage and oxidative stress activate NF-*κ*B. The heterodimeric I*κ*B kinase (IKK) promotes the degradation of I*κ*B and the release of NF-*κ*B. Moreover, NF-*κ*B translocates to the nucleus to coordinate the reprogramming of immune cells by stimulating the expression of inflammatory molecules [48]. The impact of proinflammatory factors on NF-*κ*B is accompanied by the synthesis of prostaglandins D2 and E2 (PGD2 and PGE2) activating PPAR*α* and PPAR*γ*. C-Jun-NH2-terminal kinase (JNK) and p38 mitogen-activated protein kinase (P38MAPK), which are members of the signaling pathways of mitogen-activated protein kinase (MAPK) cascades, are involved in the regulation of PPAR expression [49, 50]. Activated PPARs (all isoforms) inhibit NF-*κ*B activity, leading to the suppression of inflammation.

Possible mechanisms of NF-*κ*B inactivation include not only direct binding of PPARs but also the indirect effect of these receptors by stimulating the production of antioxidant enzymes and by reducing the concentration of reactive oxygen species involved in the pathogenesis of inflammation. It has been demonstrated that the binding of PPAR*α* and PPAR*γ* to NF-*κ*B leads to the proteolytic degradation of NF-*κ*B [5, 6]. The activation of PPAR*β*/*δ* also promotes the disruption of the function of NF-*κ*B [46]. For example, PPAR*β*/*δ* suppresses the binding of NF-*κ*B to DNA resulting in the inhibition of the transcription of NF-*κ*B target genes [37]. PPAR*α* and PPAR*γ* can inhibit the acetylation of NF-*κ*B [51]. It has been found that PPAR*α* and PPAR*γ* also enhance the expression of I*κ*B*α*, a protein of the I*κ*B family binding to NF-*κ*B in inflammatory reactions [52]. In addition, PPAR*α* activation increases the expression of sirtuin 1 (SIRT1), which inhibits NF-*κ*B [53]. The effect of PPAR*α* on SIRT1 is dependent on AMP-activated protein kinase (AMPK). AMPK activation leads to the phosphorylation of P300 resulting in the reduction of its activity. However, SIRT1 and AMPK activate each other as well. There is no accurate data on whether PPAR*α* activates SIRT1 directly or through AMPK activation [54].

Thus, PPARs significantly exert their anti-inflammatory effect through the inhibition of NF-*κ*B. At the same time, there are studies considering the proinflammatory role of PPAR*γ*, which consists in initiating the development of type 2 (or T2) inflammation [8]. PPAR*γ* is able to control Th2 effector responses in T cells and induce Th2 immunity through dendritic cells. Perhaps PPAR*γ* only performs proinflammatory functions by interacting with these cells. In addition, PPAR*γ* plays this role mainly in “pathogenic” Th2 cells. Thus, PPARG expression was increased only in CRTh2+Th2 cells that produce IL-5, in contrast to CRTh2−Th2. Inhibition of PPAR*γ* in differentiated Th2 cells decreased the secretion of type 2 cytokines. Thus, PPAR*γ* possess both anti-inflammatory and proinflammatory effects. The relationships between PPARs, NF-*κ*B, and TLRs play an important role in the signaling mechanisms involved in the development and the resolution of inflammation [55–57].

Toll-like receptors (TLR1–13) are localized on the surface of cells of the immune system (macrophages, dendritic cells, mast cells, neutrophils, basophils, natural killer cells, and B and T cells) and nonimmune cells (fibroblasts, epithelial cells, and keratinocytes). TLRs are divided into the membrane (TLR1, TLR2, TLR4, TLR5, TLR6, and TLR10) and endosomal receptors (TLR3, TLR7, TLR8, and TLR9) [58, 59].

All TLRs (except TLR3) transmit a signal through the TIR domain (toll/interleukin-1 receptor domain) to adapter molecules initiating kinases that activate proinflammatory factors, in particular, NF-*κ*B [55]. These adapter molecules include myeloid differentiation protein 88 (MyD88), TIR domain-containing adapter (TIRAP), and TIR domain-containing adapter-inducing interferon-*β* (TICAM/TRIF) 1 and 2. All TLRs exert their action through the Myd88, except for TLR3, which transmits a signal through TRIF. TLR4 activates both MyD88-dependent and TRIF-dependent signaling pathways; therefore, the receptor recognizes a significant number of ligands [60]. In addition to TLR4, TLR2 recognizes a large number of ligands [61, 62].

It is of interest that there is sufficient evidence demonstrating the two-way relationship between PPARs and TLRs [63]. Activation of TLRs is accompanied by both the hyperexpression of PPAR*β*/*δ* and the suppression of the activity of PPAR*α* and PPAR*γ* [49]. A consequence of the low expression of PPARs is an increase in the level of proinflammatory cytokines and the initiation of an inflammatory response [64]. These results reveal that the association between PPARs and TLRs regulates the inflammatory response. The signaling pathways of TLRs are involved in the activation of the proinflammatory factor NF-*κ*B, while PPARs inhibit its activity. The relationships between PPARs, NF-*κ*B, and TLRs can be the basis for the development of strategies to control the activity of the inflammatory process in asthma.

Another important mechanism of PPAR action is its ability to regulate lipid metabolism. The anti-inflammatory effect of PPARs is also realized through the effect on the metabolism of lipid mediators (by regulating the oxidative degradation of fatty acids). PPARs are involved in the control of genes responsible for oxidative lipid metabolism (carnitine palmitoyltransferase I (CPT1), pyruvate dehydrogenase lipoamide kinase isozyme 4 mitochondrial (PDK4), CYP4A, and acyl-CoA oxidase 1 (ACOX1)) [65]. Therefore, PPARs are regulators of mitochondrial and peroxisomal *β*-oxidation, ketogenesis, and bile acid synthesis. However, all isoforms of PPARs demonstrate functional differences [63]. PPAR*β*/*δ* and PPAR*α* increase energy dissipation, and PPAR*γ* promotes energy storage. PPAR*α* activates lipid metabolism during fasting, and PPAR*γ* stimulates lipogenesis during anabolism. PPAR*γ* and PPAR*β*/*δ* along raise insulin sensitivity.

PPARs are able to modulate energy homeostasis through a number of other mechanisms [66] as well. PPAR*γ* and PPAR*γ* coactivator-1*α* (PGC-1*α*) participate in the oxidative metabolism of mitochondria. Nuclear receptor-interacting protein 1 (NRIP1) binds to PPAR and inhibits the gene expression involved in energy consumption. Mediator complex subunit 1 (MED1) plays a great role in energy homeostasis by interacting with PPARs.

Thus, PPARs are able to correct lipid and immune homeostasis that is impaired under the conditions of pathology development. Therefore, these receptors can serve as therapeutic targets for the various diseases, asthma in particular.

## 5. PPAR-Dependent Mechanisms in Asthma

PPARs are involved in the regulation of inflammatory reactions and lipid metabolism, which are important components of asthma pathogenesis.

Association of genetic polymorphism of PPARs and risk of asthma development was investigated, observed, and demonstrated, but not established by Zhang et al. [54]. The correlation between rs1805192, rs10865710, and levels of IL-4 and IL-5 has been found. In addition, the parameters of the St. George's Respiratory Questionnaire (SGRQ) evaluating the quality of life have been correlated with rs1805192 and rs10865710. These results prove the importance of PPARs in asthma pathogenesis.

The importance of PPAR*γ* activation in the resolution of pneumonia has been confirmed by experimental studies [42, 67, 68]. A decrease in the proliferation of smooth muscle cells of airways in rats upon the activation of PPAR*γ* was observed [69]. Lakshmi et al. have shown that the PPAR*γ* deficiency contributes to the development of airway hyperresponsiveness, bronchial remodeling, and the inhibition of MUC5AC activity [70]. However, interpretation of PPAR*γ* level in asthma requires particulars, because this parameter can change depending on the stage of inflammation (initiation or resolution) [4]. In response to the action of the allergen, Th2 cells express PPAR*γ*, which promotes the synthesis of the IL-33 receptor on the surface of Th2 cells. This fact allows us to consider the PPAR*γ* a factor that controls immune responses of type 2 in allergy [71]. The PPAR*γ* is able to control Th2 effector responses in T cells and through dendritic cells induce Th2 immunity in pulmonary pathology [8].

PPARs inhibit the proinflammatory activity of NF-*κ*B as a major regulator of innate and adaptive immune responses in asthma [72]. This factor plays an important role in the regulation of Th2 cytokine production, mucus hyperproduction, and airway remodeling processes involved in asthma pathogenesis [73]. Sun et al. have demonstrated that metformin, which is used for the treatment of diabetes type 2, reduces lipopolysaccharide-induced damage to bronchial epithelial cells by suppressing NF-*κ*B signaling [74]. The inhibition of the NF-*κ*B signaling pathway is associated with the anti-inflammatory effect of *Physalis peruvianaL*., used in the model of allergic asthma in animals [75]. It is apparent that the study of NF-*κ*B functions is necessary to search for new therapeutic approaches in asthma.

If PPARs inhibit NF-*κ*B, then TLRs are involved in its activation and regulation of PPARs' activity. TLRs are widely expressed in cells of the respiratory tract and are on the first line of defense of the mucous membrane thereby participating in the recognition and the elimination of pathogenic microorganisms and air allergens [76]. TLRs control the synthesis of proinflammatory cytokines and proallergic mediators (TSLP, IL-25, and IL-33) that enhance the Th2-mediated response, and as a result, the processes of hyperreactivity and remodeling of the respiratory tract occur [55, 77, 78]. It should be noted that the modulation of TLR signaling can be aimed at both the activation and the resolution of airway inflammation [55, 79].

Mast cells express the majority of TLRs and are involved in the cytokine induction secretion and the chemokine-initiated Th2 immune response [80]. The activation of basophilic TLRs is accompanied by an increase in the production of IL-4, IL-8, and IL-13 [60, 77]. The stimulation of TLRs on eosinophils also leads to the release of cytokines involved in the development of the Th2 immune response [81]. In addition, TLRs induce oxidative stress and stimulate the release of growth factors and cytokines associated with airway remodeling [72].

Bacterial products facilitate the recruitment of regulatory T cells (Tregs) into the respiratory tract and the activation of dendritic cells via TLRs [82]. Some microbial agents activate TLRs and may be involved in asthma progress [83]. A distinctive property of TLRs is their participation in the development of the immune response to viral and bacterial infections that cause asthma exacerbations.

Endosomal TLRs are related to the induction of asthmatic inflammation and the development of asthma exacerbations in response to viral and bacterial infections [78]. The activation of TLR3 is a mechanism of enhancing airway inflammation upon a viral infection [84]. TLR7/8 may be responsible for asthma exacerbation during the development of viral infection [81]. The activation of TLR9 promotes the development of Th1 immune response and a decrease in the level of Th2-associated cytokines (IL-4, IL-5, IL-12, and IL-1*β*). The enhancement of Th1 immune response is likely to be the mechanism by which TLR9 inhibits the activation of the Th2 type inflammation [85]. TLR9 is involved in asthma exacerbation in viral infection [81].

The activation of receptors expressed on the cell membrane (predominantly TLR2 and TLR4) by allergens is accompanied by the formation of the Th2 immune response and the development of allergic inflammation [76]. The activation of TLR2 by agonists may lead to both inhibition of [79] and contribution [86] to asthma development. Despite the marked role of TLR4 in the induction of airway hypersensitivity [87], MyD88 expression is essential for asthma development [88].

It is important to understand the mechanism of relationship between TLRs and PPARs on the inflammatory signaling cascade for further investigation of alternative strategies for the prevention and treatment of asthma.

The role of PPARs in the regulation of lipid metabolism is well known. However, the mechanisms of the PPARs' effect on lipid homeostasis in asthma are currently being studied. In spite of the confirmed fact of the involvement of many inflammatory mediators in asthma pathogenesis, difficulties in the development of new anti-inflammatory drugs to treat this pathology still remain [89]. There are a sufficient number of PPAR activators, but their effect on each isoform is very specific and is associated with some side effects. The investigation of signaling mechanisms involved in the implementation of the anti-inflammatory effects of these nuclear factors should be done.

## 6. Natural and Synthetic Ligands of PPARs in Asthma Therapy

In the process of inflammation, a wide range of metabolites and synthetic activators stimulate PPARs, thereby modulating the proinflammatory response and preventing their excessive activation [90, 91].

### 6.1. Natural Ligands for PPARs

Various proinflammatory and proresolving lipid mediators are involved in the regulation of inflammation. The most famous natural PPAR ligands are presented in Table 1.

Natural ligands for all PPAR isoforms include eicosanoids (oxidized metabolites of FAs), PUFAs, and endocannabinoids; natural ligands for PPAR*γ* are SPMs [22, 38, 41, 92–99].

#### 6.1.1. Eicosanoids

Eicosanoids are proinflammatory lipid mediators, including the prostanoid family: PG (PG12, PGF2, PGD2, and PGE2), cyclopentenone PG (15d-PGJ2, *Δ*12-PGJ2, PGJ2, PGC2, PGA1, and PGA2), and thromboxane A2 (TxA2); the LT family (LTC4, LTD4, LTE4, LTF4, LTD4, LTA4, and LTB4); and the eoxin family (A4, C4, D4, and E4) [100]. Eicosanoids play a key role in the initiation of the acute inflammatory process [101–110] (see Table 2).

PG synthesis is the result of the metabolism of arachidonic acid, and their action results from various G-protein-related receptors that determine their immunological effects. Therefore, the same PG can have the opposite effect depending on the activated signaling pathways [107]. Most PGs have a proinflammatory effect (for example, PGE2 and PGD2), but some of them, cyclopentenone PG (PGA2 and 15d-PGJ2) in particular, are characterized by anti-inflammatory and antioxidant properties and participate in the regulation of glucose metabolism [92].

15-Deoxy-*Δ*-12,14-prostaglandin J2 (15d-PGJ2) is the first discovered endogenous PPAR*γ* ligand that exerts its effect by developing a PPRE response [44]. Heterodimers with RXR*α* receptors are formed after binding 15d-PGJ2 and PPAR*γ* together. The association of this complex with DNA results in the expression of genes encoding receptors of glucose metabolism (IRS-1 and IRS-2) and factors of inflammatory process (TNF-*α*, NF-*κ*B, and AP-1) [43]. The anti-inflammatory and antioxidant effect of 15d-PGJ2 in lung pathology has been demonstrated in a number of experimental studies and is considered to be a promising area for further investigation [108, 109].

AA metabolites can modulate not only PPAR*γ* (PGA2 and 15d-PGJ2) but also PPAR*α* (LTB4 and hydroxyeicosatetraenoic acids (HETEs): 5(S)-HETE, 8(S)-HETE, 15-HETE, and 8S-HPETE) [41]. Besides, prostacyclin and 15-HETE can activate PPAR*β*/*δ* [38]. However, 5(S)-HETE, 8(S)-HETE, 15-HETE, and LTB4 are weaker activators [94]. It is worth noting that the functioning of NK cells, which play a key role in limiting allergic inflammation in asthma, is regulated by eicosanoids including PGD2 and PGE2.

The production of PGE2 and PGD2 is known to increase in the inflammation; they are transformed into anti-inflammatory PGA2 and 15d-PGJ2 [94]. Besides, the resolution of acute inflammation is the process of switching the synthesis of proinflammatory lipid mediators (PG, LT) to the formation of endogenous SPMs [110]. The precursors of SPMs are PUFAs.

#### 6.1.2. PUFAs

PUFAs have been shown to exert an anti-inflammatory effect through PPAR activation [38, 111]. All PPARs have a similar structure due to the presence of a ligand-binding domain, which provides their activation by FAs and FA derivatives consisting of 14 and more carbon atoms [34].

The oxidized forms of docosahexaenoic (DHA) and eicosapentaenoic acid (EPA) can also be considered a new and effective PPAR ligand class. It has been proven that oxidized forms of EPA and low-density lipoproteins (LDL) activate PPAR*α* more efficiently than nonoxidized ЕРА and LDL, especially in endothelial cells. The investigation in this field may be of importance in creating new and effective PPAR*α* agonists. The oxidized form of DHA 17-oxodocosahexaenoic acid (17-oxoDHA) is a dual PPAR*γ*/PPAR*α* agonist [93]. Oxidized DHA derivatives are more effective in relation to PPAR*γ* as compared to synthetic PPAR ligands.

The role of nutritional fatty acids in the prevention and treatment of asthma has been recently investigated [112]. The treatment of asthma by omega-3 fatty acids is more effective than sublingual immunotherapy to reduce the level of IL-17A. Both therapeutic methods have been shown to effectively reduce the asthma control test (ACT), peak expiratory flow rate (PEFR), and forced expiratory volume in the first second (FEV1) [113]. However, the lack of standardized doses significantly limits the recommendations for their use [114]. The question whether the dietary intake of fatty acids protects against asthma still remains controversial. Despite the favorable results of the use of omega-3 fatty acids for the treatment of asthma, the European Prospective Investigation into Cancer and Nutrition (EPIC) Heidelberg cohort study has not demonstrated a protective effect of alimentary fatty acids in asthma [115]. In addition, fish and fatty acid consumption during pregnancy is related to the development of asthma in newborn children [112].

The violation of the FA composition of cell membranes and the imbalance between substrates for the synthesis of pro- and anti-inflammatory mediators are two of the major factors for promoting the development and aggravation of chronic respiratory diseases, including asthma [24, 25].

#### 6.1.3. SPM

In both AA and DHA, EPA can act as precursors for the production of SPMs involved in the resolution of inflammation, which include Rvs (RvE (RvE1, RvE2), RvD (RvD1, RvD2, RvD3, RvD4, RvD5, and RvD6), and RvT (RvT1, RvT2, RvT3, and RvT4)), LXs (LXA4, LXВ4), PDs (РD1), and MaRs (МaR1, MaR2) [110, 116]. Proresolving lipid mediators and their lung functions are presented in Table 3.

According to Muralikumar et al., the highest active PPAR*γ* agonists among FAs and their derivatives are RvE1, PD1, DHA, EPA, and LXA4 (listed in descending order of their activity) [34]. DHA and EPA as PPAR*γ* agonists have been reviewed above.

*(1) Resolvins*. Rvs are the main humoral factors contributing to the resolution of inflammation [117]. Rvs of the E-series (RvE1, RvE2) are formed from EPA, and Rvs of the D-series (RvD1, RvD2, RvD3, RvD4, RvD5, and RvD6) are produced from DHA [116]. The anti-inflammatory properties of RvE1 result from the interaction with PPARs and NF-*κ*B blocking. RvE1 receptors include leukotriene B4 receptor 1 (BLT1) and G protein-coupled receptor-chemerin receptor 23 (ChemR23). RvE1 has an antagonistic effect on BLT1 by blocking the biological properties of proinflammatory LTs. Nevertheless, RvE1 has a synergistic effect on ChemR23, blocks NF-*κ*B activation, and enhances phagocytosis. The powerful protective action of RvE1 has been demonstrated in the inflammation of the respiratory tract, namely, in asthma. RvE1 has been shown to reduce airway hyperreactivity in mice [118]. RvE2 has a biological effect that is similar to that of RvE1; it regulates neutrophil chemotaxis and activates phagocytosis and synthesis of anti-inflammatory cytokines. The anti-inflammatory mechanism of RvD action in bronchopulmonary pathology is under study [119, 120]. Lipoxin A4 receptor (ALX) and G protein-coupled receptor 32 (GPR32) have been identified as receptors for RvD1. The experiments have revealed the ability of RvD1 to reduce the production of proinflammatory cytokines and airway hyperreactivity [119, 121].

*(2) Protectins*. PDs formed from DHA are synthesized by a number of cells, including brain cells, monocytes, and CD4+ lymphocytes [122]. PD1 (the key member of the PD family) has an anti-inflammatory and neuroprotective effect. The action of this mediator is realized by blocking NF-*κ*B and decreasing COX-2 expression and PG synthesis. The ability of PD1 to inhibit 15-lipoxygenase (15-LOX) expression and thereby LT biosynthesis has been shown [123]. PD1 is a regulator of the synthesis of proteins of the B cell lymphoma 2 (BCL2) family, which have a pronounced antiapoptotic effect [116]. The decrease in PD1 has been found in severe and uncontrolled asthma [124]. Recently, PD1 has been identified in the exhaled condensate of asthma patients during exacerbation. In addition, PD1 reduces the level of PGD2 involved in the development of airway hyperresponsiveness. It has been established that intravenous injection of PD1 into allergen-sensitive mice before the administration of an aerosol allergen protects animals from the development of airway hyperreactivity, as well as eosinophilic and T cell-mediated inflammation [123].

*(3) Lipoxins*. LXs, powerful anti-inflammatory bioregulators suppressing the inflammatory process and activating resolution and recovery processes, are PPAR agonists [125]. LXA4 protects cells from damage by activating the p38 MAPK/PPAR*γ*/nuclear factor E2-related factor 2-antioxidant-responsive element/heme oxygenase 1 pathway [126]. Impaired LX production is associated with asthma pathogenesis [127]. LXA4 is an endogenous mediator of inflammation of the mucous membrane, and it reduces the severity of allergic and asthmatic reactions. The decrease in the LXA4 level in exhaled condensate correlates with lung function impairment. The investigation by Larsson et al. has revealed differences in the level of LXs in bronchial wash fluid and bronchoalveolar lavage fluid [128]. LX concentration in bronchial wash fluid was higher in the group of asthma patients compared to the control group, and its level in bronchoalveolar lavage fluid did not differ between these groups. The revealed violations of LX production in asthma suggest that these mediators can be of significant interest as a therapeutic target in the disease.

The study of proresolving lipid mediators, which are PPAR agonists, opens up new ways for the development of an effective strategy for asthma treatment by correcting the processes of the inflammation resolution [110, 129].

#### 6.1.4. Endocannabinoids

The endocannabinoid system is a universal signaling system performing important functions, such as the regulation of energy balance, metabolism of carbohydrates and lipids, the participation in the development of immune response, and the suppression of inflammatory processes by inducing Treg apoptosis and inhibiting cell proliferation and production of proinflammatory mediators [130]. The system includes endocannabinoids (N-arachidonoylethanolamide (AEA or anandamide) and 2-arachidonoylglycerol (2-AG)), cannabinoid receptors (CB1 and CB2 receptors), and enzymatic systems involved in their synthesis, transport, metabolism, and degradation [131].

Endocannabinoids interact primarily with cannabinoid receptors CB1 and CB2. The CB1 receptor has been found in the central and peripheral nervous system, lungs, kidneys, and liver; the CB2 receptor is expressed by immune and hematopoietic cells. Recent evidence points out that endocannabinoids interact with not only these receptors but also G protein-coupled receptor 55 (GPR55) and all PPAR isoforms [96, 132, 133]. For example, it has been shown that endocannabinoids can serve as double agonists of PPAR*γ* and CB2 and neutralize chronic inflammation [95]. It has been observed that endocannabinoids and cannabinoid-like molecules and some of their metabolites activate PPARs [97]. Thus, endocannabinoid AEA, as well as endocannabinoid-like compounds, such as N-docosahexaenoylethanolamine (DHEA), N-eicosapentaenoylethanolamine (EPEA), N-oleoylethanolamine (OEA), N-palmitoylethanolamine (PEA), and N-stearoylethanolamine (SEA), activate PPAR*α* [97]. Furthermore, 15D-PGJ2-glycerol ester (a metabolite of 2-AG) exerts an anti-inflammatory effect through PPAR*γ* activation [98]. Anandamide and PEA are involved in the physiological mechanisms of vascular regulation through PPAR*α* activation [97]. The effect of endocannabinoids on PPAR*β*/*δ* has been described in certain studies. Yu et al. have shown that anandamide initiates PPAR*β*/*δ* activation [99]. There is little research on synthetic cannabinoids activating PPARs. There is evidence that WIN55 212-2, andarachidonyl-2′-chloroethylamide, CP55940, HU331, and JWH015 have impact on PPAR*α* and PPAR*γ* activity [134].

Endocannabinoid receptors, like PPARs, are expressed by alveolar macrophages, eosinophils, monocytes, and dendritic cells [42, 135]. The receptors PPAR*α*, CB1, and CB2 that directly interact with endocannabinoids [132] are located in the bronchi of mice [136]. The endocannabinoid 2-AG and its receptor CB2 play an important role in the inhibition of mast cells (MC), as well as in the migration of eosinophils to the respiratory tract [137]. Endocannabinoids inhibit cytokine production by lung NK cells through the interaction with CB2 receptors. Ferrini et al. suggest that NK cells limit ILC2 responses during allergic airway inflammation [138]. Mature ILC2 is known to produce Th2-type cytokines (IL-4, IL-5, IL-9, and IL-13) involved in the initiation of an adaptive immune response in asthma. Mast cells and eosinophils express the majority of TLRs and induce the secretion of cytokines and chemokines initiating Th2 immune response [80, 81]. TLR ligands stimulate macrophages and affect their production of endocannabinoids, whose function is to suppress TLR-mediated inflammatory response [139, 140]. The effect of cannabinoids on macrophage function is a consequence of NF-*κ*B inhibition [141]. Thus, endocannabinoids act through cannabinoid receptors and PPARs regulating the activity of TLRs and NF-*κ*B [142].

The role of the endocannabinoid system in lung pathophysiology may be of interest compared to studying the anti-inflammatory function of synthetic cannabinoids in asthma. A number of studies have shown that oral or aerosol administration of cannabinoids leads to a bronchodilating effect in asthma patients. Recently, selective agonists and antagonists for the CB1 and CB2 receptors have been developed, and their effectiveness has been demonstrated in the experimental model of asthma [143]. The data obtained reveal the therapeutic potential of cannabinoids as PPAR agonists in asthma and promote further studies in this field. Though the effectiveness of synthetic cannabinoids has been established, their therapeutic significance is worth discussing [139, 144]. The limitation of clinical use of cannabinoids in a pain treatment has side effects (drowsiness, dizziness, speech impairment, memory impairment, and mental confusion) caused by therapeutically active doses of the agent. The conflicting effects observed in clinical trials of synthetic cannabinoids are likely to be related to the heterogeneity of their receptors and the complexity of cannabinoid signaling. Cannabinoid derivatives that target mainly CB2 receptors may be the most promising drugs due to the lack of central side effects [143].

### 6.2. Synthetic Ligands for PPARs

The most famous synthetic PPAR ligands are presented in Table 4.

Synthetic ligands for PPAR*α* are fibrates used in treating hypertriglyceridemia (the effectiveness of the use of fibrates is rather low for other PPAR isoforms); PPAR*β*/*δ* synthetic ligands are GW501516, GW0742, and L-165041; PPAR*γ* synthetic ligands are thiazolidinediones widely used for the treatment of diabetes and having pronounced anti-inflammatory properties [33, 37, 38, 46, 89, 145]. There is evidence of the effectiveness of using thiazolidinediones as ligands for other PPAR isoforms [90, 146].

#### 6.2.1. Fibrates

Fibrates are synthetic ligands for PPARs and are used in the treatment of hypertriglyceridemia. It is known that PPARs regulate the concentration of lipids, lipoproteins, and glucose in blood. They participate in the pathogenesis of obesity as well. Fibrates are synthetic ligands mainly for PPAR*α* (clofibrate, fenofibrate, bezafibrate, and pemafibrate), while bezafibrate activates other PPAR isoforms, but with lower efficiency [33]. Fibrates, like PUFAs, stimulate the metabolism of dietary FAs and affect the metabolism of triglycerides via PPAR*α* [145]. Synthetic ligands for PPAR*γ* are fibrates such as clofibrate, fenofibrate, gemfibrozil, and ciprofibrate [145]. Fibrate metabolites (clofibric and fenofibric acids) are dual activators of PPARa/*γ*. Fibrate therapy, however, can cause a number of adverse effects, for example, an increase in serum creatinine levels. Possible side effects of these compounds require further studying.

The combined use of fenofibrate and dexamethasone leads to the suppression of the production of IL-23 and IL-1 in rats with asthma. This is evidence of the effectiveness of the combined therapeutic regimens in asthma [12]. The opportunity of using fenofibrate both in therapy and the prevention of bronchial remodeling in asthma has been shown [147]. Fibrates as therapeutic agents in asthma are currently being studied. As is known, they are much used for the treatment of obesity, so fibrates can be also applied to the therapy of asthma related to obesity [148]. This field needs further investigation.

#### 6.2.2. Thiazolidinediones

Thiazolidinediones are synthetic ligands mainly for PPAR*γ*, and yet there is evidence of their effectiveness as ligands for other PPAR isoforms (for example, lobeglitazone sulfate is a dual PPAR*α*/*γ* agonist) [146]. Recent experimental data confirm the clinical advantages of thiazolidinediones in asthma treatment [89]. In addition to improving lung function, these drugs reduce the risk of asthma exacerbation and the need for oral administration of steroids [13]. However, the use of thiazolidinediones for asthma treatment is accompanied by side effects. This problem is to be studied more thoroughly.

The experimental study has shown that the rosiglitazone reduces the hyperreactivity of the respiratory tract [149]. The authors of the study have established that rosiglitazone inhibits the secretion of Th2 cytokines, which are involved in the inflammatory response of the respiratory tract in asthma. Its significant efficiency in asthma has been established in course placebo-controlled randomized trials (asthma patients received rosiglitazone for 4 weeks) [150]. In addition, the use of rosiglitazone is accompanied by an increase in body weight and edema.

Troglitazone demonstrates anti-inflammatory properties in asthma that are the result of inhibition of the synthesis of LTC4, one of the proinflammatory lipid mediators causing bronchoconstriction, hyperreactivity, and edema of the bronchi [151]. However, troglitazone is associated with hepatotoxicity, so its clinical trials have been terminated.

Pioglitazone reduces the inflammation and bronchial hyperreactivity in asthma [129]. It is effective in patients who do not receive the modern combination of corticosteroids and *β*2-agonists [10]. Nevertheless, Kaler et al. have highlighted that the use of pioglitazone for severe asthma therapy is unsafe since 14% of patients had serious side effects caused by the discontinuation of the drug [152]. Anderson et al. did not find any signs of improvement in the cause of the disease after a 12-week treatment with pioglitazone [68]. Besides, the use of pioglitazone is accompanied by an increase in body weight and the risk of fractures, which, in turn, is the sign to continuous studying of this drug.

Ciglitazone (synthetic PPAR*γ* agonist) suppresses the development of bronchial remodeling, airway hyperresponsiveness, and mucus hypersecretion [153]. Ciglitazone has been shown to be able to modulate the expression of the intercellular adhesion molecule 1 (ICAM-1) gene in smooth muscle cells of the respiratory tract, and this gene initiates the production of proinflammatory cytokines (TNF*α*). It is known that proinflammatory cytokines regulate the function of smooth muscle cells of the respiratory tract and thereby contribute to the development of bronchial hyperreactivity in asthma.

Another PPAR agonist, namely, 4-hydroxy-12-(4-hydroxyphenethyl) isoindoline-1,3-dione, is a promising agent in asthma treatment. The experiment has revealed a decrease in the severity of inflammation and mucin secretion in the lungs of mice treated with this agonist [154].

PPAR agonists exhibit an anti-inflammatory effect by acting on proinflammatory mediators and cells related to asthma pathophysiology [4]. Maslanka et al. have reported that PPAR*γ* agonists are not able to exert a direct inhibitory effect on the production of IL-4, IL-10, and IL-17 by T helper cells (CD4+ cells) in asthma treatment. In addition, Tregs are not involved in the realization of the antiasthmatic effect of PPAR*γ* agonists [155].

Thus, a wide range of metabolites and agents can activate PPARs during the development of inflammatory response, but their effect on each of the isoforms is very specific. The association between PPAR isoforms and lipid metabolism as well as their high affinity for PUFAs, endocannabinoids, and eicosanoids, has been established [156]. Moreover, PUFAs are precursors for the formation of endogenous specialized proresolving lipid mediators (RvE1, PD1, and LXA4), which are highly active PPAR*γ* agonists. Fibrates are synthetic ligands for all PPAR isoforms with weakly pronounced activity for PPAR*β*/*δ* and PPAR*γ*. PPAR*γ* exhibits high affinity for thiazolidinediones.

Therefore, we can conclude that the investigation of PPARs is an extremely promising area. There are many unresolved issues in the investigation of anti-inflammatory signaling pathways.

## 7. Conclusion

Asthma is a chronic, heterogeneous inflammatory disease with complex etiopathogenesis and a progressive course that is related to type 2 inflammation. In response to the action of allergens, early type 2 immune response enhances, which, in turn, results in the activation of bronchial epithelial cells and the production of IL-33, IL-25, and TSLP.

Modulators of the inflammation activity and lipid homeostasis are PPARs, which are important components of asthma pathogenesis. The present-day understanding of PPARs primarily indicates the anti-inflammatory potential of this receptor, which exerts its anti-inflammatory effect through the inhibition of NF-*κ*B. At the same time, TLR signaling pathways activate NF-*κ*B. The crosstalk between PPARs, TLRs, and NF-*κ*B is of great importance in the pathogenesis of the inflammatory process in asthma. The predominance of proinflammatory signaling with TLRs and NF-*κ*B contributes to chronic inflammation and asthma impairment. Therefore, special attention is focused on the activation of anti-inflammatory PPAR signaling.

Studies that examine the proinflammatory role of PPARs, in particular PPAR*γ*, are limited. PPAR*γ* is taken as a mediator of interactions between dendritic and T cells in type 2 (or T2) inflammation, thereby initiating the development of type 2 (or T2) inflammation. In addition, PPAR*γ* plays this role mainly in “pathogenic” Th2 cells. Thus, PPAR*γ* expression was increased only in CRTh2+Th2 cells that produce IL-5, in contrast to CRTh2−Th2. Inhibition of PPAR*γ* in differentiated Th2 cells decreased the secretion of type 2 cytokines. The ambiguity of the signaling mechanisms of PPAR isoforms requires further in-depth study.

There are a sufficient number of activators of PPARs, but their effect on each of the isoforms of these receptors is very specific. PPARs have a high affinity for PUFAs, endocannabinoids, and eicosanoids. Synthetic ligands for PPARs are fibrates, but their activity is less pronounced for PPAR*β*/*δ* and PPAR*γ*. Thiazolidinediones are synthetic ligands primarily for PPAR*γ*. PUFAs are precursors for the formation of endogenous specialized proresolving lipid mediators (RvE1, PD1, and LXA4), which are highly active PPAR*γ* agonists.

Further studies of the mechanisms of influence and the combined use of natural and synthetic ligands of PPARs have great potential. Regulation of PPARs will help control the process of chronic inflammation by inhibiting the signaling mechanisms of NF-*κ*B and TLRs. The ultimate goal of future research is to obtain new fundamental knowledge about the molecular mechanisms of PPAR activation, promoting the prevention and treatment of asthma.

## Figures and Tables

**Table 1:** Natural ligands for PPARs.

Ligands	PPAR*α*	PPAR*β*/*δ*	PPAR*γ*	Reference
Eicosanoids (PG)	LTB4, 8 (S)-HETE	8 (S)-HETE	8 (S)-HETE, 15-HETE, PGA1, PGA2, PGD2, 15d-PGJ2	[41, 43, 44, 92]
PUFAs, SPM	АА, EPA, LDL, DHA, 17-oxoDHA	АА, EPA, DHA	АА, EPA, DHA, 17-oxoDHA, RvE1, PD1, LXA4	[38, 93, 94]
Endocannabinoids	Anandamide, 2-AG, OEA, PEA	Anandamide	Anandamide, 2-AG, 15D-PGJ2-glycerol ether	[95–99]

**Table 2:** Proinflammatory lipid mediators and their functions.

Mediator		Function	Reference
LT family	LTC4	Bronchoconstriction	[101–104]
	LTD4	Bronchoconstriction	
	LТЕ4	Bronchoconstriction	
	LTF4	—	
	LTA4	—	
	LTB4	Mediates chemotaxis, plasma exudation, reduction of lung parenchyma	

Prostanoid family	PGI2	Vasodilation, inhibitory effect on platelet aggregation	[43, 44, 92, 94, 105–107, 109, 110]
	PGE2	Bronchoconstriction, bronchodilation, activation of autonomic neurotransmitter pyrogenic hyperalgesia	
	РGF2	Bronchoconstriction	
	PGD2	Bronchoconstriction	
	15d-PGJ2	Antioxidant effect	
	TxA2	Vasoconstriction	
Eoxin family	A4, C4, D4, E4	The development of allergy	

**Table 3:** Proresolving lipid mediators and their lung functions.

Mediator		Function	Reference
MaRs	МaR1	Reduces expression of IL-5 and IL-13, decreases lung neutrophils, tissue hypoxia	[117]
Rvs	RvE1	Decreases in airway hyperresponsiveness, regulation of neutrophil chemotaxis, activation of phagocytosis, and synthesis of anti-inflammatory cytokines	[117–121]
	RvD1	Reduces synthesis of proinflammatory mediators and eosinophilia	
РDs	РD1	Decreases airway hyperresponsiveness	[116, 122–124]
LXs	LXA4	Slows down chemotaxis and migration to the area of inflammation of macrophages and neutrophils, blocks lipid peroxidation, activates NF-*κ*B, and inhibits the synthesis of proinflammatory cytokines	[117, 125–128]

**Table 4:** Synthetic ligands for PPARs.

Ligands	PPAR*α*	PPAR*β*/*δ*	PPAR*γ*	Reference
Fibrates	Bezafibrate, clofibrate, fenofibrate, pemafibrate	Bezafibrate	Clofibrate, fenofibrate, gemfibrozil, ciprofibrate	[33, 145]
Thiazolidinediones	Lobeglitazone sulfate		Rosiglitazone, siglitazone, pioglitazone, 4-hydroxy-12-(4-hydroxyphenethyl) isoindoline-1,3-dione, troglitazone, lobeglitazone sulfate	[33, 37, 38, 46, 89, 90, 145, 146]

## Abbreviations

AA: Arachidonic acid
ACOX1: Acyl-CoA oxidase
AEA: N-Arachidonoylethanolamide
ALX: Lipoxin A4 receptor
AMPK: AMP-activated protein kinase
AP-1: Activator protein-1
ACT: Asthma control test
BLT1: Leukotriene B4 receptor 1
BCL2: B cell lymphoma 2
CB1, CB2: Cannabinoid receptors
CD4+ cells: T helper cells
ChemR23: Chemerin receptor 23
COX-2: Cyclooxygenase-2
CPT1: Carnitine palmitoyltransferase I
C/EBP: CCAAT/enhancer-binding protein
DHA: Docosahexaenoic acid
DHEA: N-Docosahexaenoylethanolamine
EPA: Eicosapentaenoic acid
EPEA: N-Eicosapentaenoylethanolamine
FAs: Fatty acids
FEV1: Forced expiratory volume in the first second
GPR32: G protein-coupled receptor 32
GPR55: G protein-coupled receptor 55
HETEs: Hydroxyeicosatetraenoic acids
HO-1: Heme oxygenase 1
IKK: Heterodimeric I*κ*B kinase
I*κ*B: NF-*κ*B inhibitor protein
IL: Interleukin
IL-1R: Interleukin-1 receptor
ILC2: Type 2 innate lymphoid cells
iNOS: Inducible nitric oxide synthase
ICAM-1: Intercellular adhesion molecule 1
IRS-1, IRS-2: Insulin-1 and insulin-2 receptors
LBD: Ligand-binding domain
LDL: Low-density lipoproteins
LT: Leukotriene
LXs: Lipoxins
MAPK: Mitogen-activated protein kinase
MaRs: Maresins
MC: Mast cells
MED1: Mediator complex subunit 1
MMPs: Matrix metalloproteinases
MyD88: Myeloid differentiation protein 88
NF-*κ*B: Nuclear factor kappa-light-chain-enhancer of activated B cells
NK cells: Natural killer cells
NO: Nitric oxide
NRIP1: Nuclear receptor-interacting protein 1
OEA: N-Oleoylethanolamine
PDs: Protectins
PDK4: Pyruvate dehydrogenase lipoamide kinase isozyme 4 mitochondrial
PEA: N-Palmitoylethanolamine
PEFR: Peak expiratory flow rate
PG: Prostaglandin
PGC-1*α*: PPAR*γ* coactivator-1*α*
P38MAPK: p38 mitogen-activated protein kinase
PPARs: Peroxisome proliferator-activated receptors
PUFAs: Polyunsaturated fatty acids
RXR*α*: Retinoid X receptor *α*
Rvs: Resolvins
SEA: N-Stearoylethanolamine
SGRQ: St George's Respiratory Questionnaire
SIRT1: Sirtuin 1
SPMs: Endogenous specialized proresolving mediators
STAT: Signal transducers and activators of transcription
TICAM/TRIF: TIR domain-containing adapter-inducing interferon-*β*
TIRAP: TIR domain-containing adapter
Th1: T helper 1 cells
Th2: T helper 2 cells
Th17: T helper 17 cells
TLRs: Toll-like receptors
TNFR: Tumor necrosis factor receptors
TNF*α*: Tumor necrosis factor *α*
Tregs: Regulatory T cells
TSLP: Thymic stromal lymphopoietin
Тх: Thromboxane
TxA2: Thromboxane A2
2-AG: 2-Arachidonoylglycerol
15d-PGJ2: 15-Deoxy-*Δ*-12,14-prostaglandin J2
15-LOX: 15-Lipoxygenase
17-oxoDHA: 17-Oxodocosahexaenoic acid.
